# Electrospun Fibers Loaded with Pirfenidone: An Innovative Approach for Scar Modulation in Complex Wounds

**DOI:** 10.3390/polym15204045

**Published:** 2023-10-10

**Authors:** Erika Maria Tottoli, Laura Benedetti, Federica Riva, Enrica Chiesa, Silvia Pisani, Giovanna Bruni, Ida Genta, Bice Conti, Gabriele Ceccarelli, Rossella Dorati

**Affiliations:** 1 Department of Drug Sciences, University of Pavia, 27100 Pavia, Italy; erikamaria.tottoli01@universitadipavia.it (E.M.T.); enrica.chiesa@unipv.it (E.C.); silvia.pisani@unipv.it (S.P.); ida.genta@unipv.it (I.G.); bice.conti@unipv.it (B.C.); 2 Department of Public Health, Experimental Medicine and Forensic, Human Anatomy Unit, University of Pavia, 27100 Pavia, Italy; laura.benedetti@unipv.it (L.B.); gabriele.ceccarelli@unipv.it (G.C.); 3 CHT Center for Health Technologies, University of Pavia, 27100 Pavia, Italy; 4 Department of Public Health, Experimental Medicine and Forensic, Histology and Embryology Unit, University of Pavia, 27100 Pavia, Italy; federica.riva01@unipv.it; 5 Physical-Chemistry Section, Department of Chemistry, University of Pavia, 27100 Pavia, Italy; giovanna.bruni@unipv.it

**Keywords:** hypertrophic scar, electrospinning, wound healing, complex wounds

## Abstract

Hypertrophic scars (HTSs) are pathological structures resulting from chronic inflammation during the wound healing process, particularly in complex injuries like burns. The aim of this work is to propose Biofiber PF (biodegradable fiber loaded with Pirfenidone 1.5 *w*/*w*), an electrospun advanced dressing, as a solution for HTSs treatment in complex wounds. Biofiber has a 3-day antifibrotic action to modulate the fibrotic process and enhance physiological healing. Its electrospun structure consists of regular well-interconnected Poly-L-lactide-co-poly-ε-caprolactone (PLA-PCL) fibers (size 2.83 ± 0.46 µm) loaded with Pirfenidone (PF, 1.5% *w*/*w*), an antifibrotic agent. The textured matrix promotes the exudate balance through mild hydrophobic wettability behavior (109.3 ± 2.3°), and an appropriate equilibrium between the absorbency % (610.2 ± 171.54%) and the moisture vapor transmission rate (0.027 ± 0.036 g/min). Through its finer mechanical properties, Biofiber PF is conformable to the wound area, promoting movement and tissue oxygenation. These features also enhance the excellent elongation (>500%) and tenacity, both in dry and wet conditions. The ancillary antifibrotic action of PF on hypertrophic scar fibroblast (HSF) for 3 days downregulates the cell proliferation over time and modulates the gene expression of transforming growth factor β1 (*TGF-β1*) and α-smooth muscle actin (*α-SMA*) at 48–72 h. After 6 days of treatment, a decrement of α-SMA protein levels was detected, proving the potential of biofiber as a valid therapeutic treatment for HTSs in an established wound healing process.

## 1. Introduction

When the skin is seriously damaged, a physiological mending response called scarring takes place. A perfect, normal, fine-line scar should ideally be flat, barely perceptible, and have neutral coloring. However, under rare circumstances, excessive scarring can result in the production of hypertrophic scars (HTSs), a pathological spectrum of scarring with a red, elevated, and bulging shape [1]. These aberrant scars can substantially impair a patient’s quality of life and physiological status by causing joint contractures, discomfort, and itching. It is difficult to comprehend the pathophysiology and create effective treatments based on scientific evidence because there are no standardized models for hypertrophic scars, even though there are a variety of therapy alternatives [2]. Although the specific etiology of hypertrophic scarring is unknown, several factors, including the type and extent of the injury, are thought to have a role. Due to a protracted and elevated inflammatory response, dysregulation in the wound healing cascade, and an excess of collagen at the site of the lesion, hypertrophic scars are likely to develop following burns and surgical treatments [3].

Therapeutic treatments for HTSs can be categorized based on their method of action [4]. Cryotherapy and ablative laser therapy destroy damaged cells, while surgical treatments like surgical excision and laser surgery remove the fibrotic tissue in an invasive way. Some treatments reduce inflammation, such as corticosteroid injections, laser therapy, pressure therapy, silicone gel, and sheets [5]. Another treatment choice is combination therapy. The patient’s unique situation and the extent of the scar also influence the treatment option. Unfortunately, most of the time, the aforementioned conventional treatments are invasive, uncomfortable for patients, and expensive for wound care procedures [6].

Many documents on wound care have been discovered, but few of them have addressed scarless healing or scar minimization [7]. It has been observed that pharmaceutical medications including both conventional plant-based components and proteins are efficient wound remedies to lessen or eliminate scarring [8]. However, it should be highlighted that to effectively treat wounds, these pharmaceutical medications must be utilized in combination with dressing or scaffolding biomaterials. The most recent recommendations for scar management suggest using sophisticated wound dressings for hypertrophic scars that are non-invasive and preventative, such as silicone sheets, polyurethanes, and alginates. Modern wound dressings are an appealing substitute for more invasive therapies since they are simple to apply and have few adverse effects, including itching, contact dermatitis, and dry skin [9].

A promising strategy for achieving most of the ideal qualities of a wound dressing in the context of advanced dressings is to use micro- and nano-fiber dressings [10]. Due to the fiber-random conformation of electrospun dressings, they can imitate the extracellular matrix and control cellular responses such as proliferation, migration, and differentiation in the skin. This characteristic can dramatically alter how long wounds take to heal. This characteristic can drastically alter the rate at which wounds heal and to expedite physiological healing, even in complex wounds [11,12]. Polymeric electrospun dressings have undergone considerable investigation in the field of wound care, and the results have been published in several review articles. There is potential for the encapsulation of several anti-scarring and antimicrobial treatments due to the variety of electrospinning nanofiber production pathways (blending, core/shell, attachment [13,14]. After using the nanofiber dressings to heal the lesions, it was stated that no burn marks could be seen 24 h after the incident. The fact that inflammatory cytokines *IL-6*, *IL-1B*, and *TNF-α* were downregulated after 24 h relative to untreated controls further supported this quick recovery [15]. Su C. et al. demonstrated that an electrospun fibrous composite membrane can speed up wound repair and prevent the formation of scars, according to an in vivo examination of wound healing and scar inhibition. Moreover, a nano-fibrous scaffold prevented microbial infiltration, kept moisture and gaseous exchange in check, and provided a high-surface-area microporous skeletal framework for rapid cell proliferation and granulation [16,17]. Overall, further study is required to fully understand the potential of electrospun dressings as a novel and efficient method of wound treatment [12,18].

The aim of this study is to evaluate the efficacy of Biofiber PF, a novel non-invasive method in the field of advanced medicated dressings for difficult wounds, to investigate a new technology that may change the treatment of hypertrophic scars. Previous research has demonstrated that Biofiber is a cutting-edge biodegradable electrospun dressing that not only controls exudate effectively, but also allows for physiological repair [17]. In this study, Pirfenidone (PF), an antifibrotic drug well-known for treating idiopathic pulmonary fibrosis [19], was encapsulated into the polymer fibers to treat pre-existing hypertrophic scars. PF has been selected on the basis of research demonstrating its impressive biological effects, including its capacity to inhibit cell proliferation, reduce inflammation, and control the fibrotic process by modulating the p38 pathway, transforming growth factor 1 (*TGF-ꞵ1*), collagen type I alpha 1 chain (*COL1A1*), and smooth muscle actin (*α-SMA*) [20,21,22].

## 2. Materials and Methods

### 2.1. Materials

Copolymer poly(Lactide-co-caprolactone), PLA-PCL 70:30 (Resomer LC 703 S Esther, Mw 160 kDa), was purchased by Evonik Industries (Evonik Nutrition & Care GmbH, Damstadt, Germany). Pirfenidone (PF) C_12_H_11_NO (Sigma Aldrich, Milan, Italy), methylene chloride (MC, C_3_H_7_NO), tetrahydrofuran (THF, C_4_H_8_O, with 250 ppm BHT (analytical grade)) were supplied by Carlo Erba (Milan, Italy). Thiazolyl blue tetrazolium bromide (MTT), phosphate-buffered saline tablet (PBS), and dimethyl sulfoxide (DMSO, C_2_H_6_OS) were obtained from Sigma Aldrich (St. Louis, MO, USA). Maximum recovery diluent (MRD), trypan blue solution (TB), Dulbecco’s phosphate-buffered saline (DPBS) 10× and Tripsyn-EDTA solution 0.25% were purchased by Sigma Aldrich, Milan, Italy. Fetal bovine serum (FBS) was furnished by Immunological Sciences, Rome, Italy DMEM High glucose w/L-glutamine (w/sodium pyruvate) was obtained from Microgem Laboratory research. Normal human dermal fibroblasts (NHDF, #LOCC2511; Euroclone S.p.A, Pero, Italy), hypertrophic scar-derived fibroblasts human (HSF, #HSF110 Lt Cheek; CellResearch Corporation, Singapore), and a cell cultures of human adult fibroblasts (p.4) was supplied by International PBI, Milan, Italy. Double-distilled water was filtered using 0.22 m Millipore Membrane filters, Millipore Corporation, Burlington, MA, USA. All chemicals which were used were of analytical grade.

### 2.2. Methods

#### 2.2.1. Preparation of Textured Dressing

PLA-PCL 70:30 was dissolved in DCM (20% *w*/*v*), and the system was kept in an ice bath overnight with magnetic stirring at 100 rpm. Pirfenidone (PF) was solubilized in DMF (8% *v*/*v*) in an ice bath for 1 h with magnetic stirring. Drop by drop, the PF solution was added to the PLA:PCL solution, which was maintained in an ice bath for 1 h with gentle magnetic stirring.

Textured fiber dressings were fabricated using electrospinning Nanon-01A (MEEC Instruments, ltd., Ogorishi, Fukuoka, Japan) in accordance with WO2021064673 [23]. The process was carried out at 35 ± 2 °C, RH 25 ± 5%. The following electrospinning settings were set: spinneret speed and width (100 mm/s and 80 mm), cleaning frequency (30 s), voltage (20 kV), flow rate (0.6 mL/h), nozzle diameter (18 G), and electrospinning time (20 min). The electrospun texturized dressing prototypes were formulated utilizing an aluminum wire conductive plate placed with a specified circular texture that was placed on the collector. The conductive plate had a circular texture, a conductive plate surface of 225 cm^2^, a dimension mesh of 1.41 mm, empty spaces #1548, and a pore area of 892.7 m^2^ [18].

#### 2.2.2. Advanced Dressing Characterization

A comparative analysis of Biofiber PF and placebo (PL) samples to a non-textured electrospun dressing as in-house control (1P), as well as two commercially available advanced dressings, Mepilex Lite^®^ (Mölnlycke Health Care AB, Goteborg, Sweden) and Biatain^®^ Alginate (Coloplast S.p.A, Bologna, Italy), was performed. Mepilex Lite^®^ is a dressing crafted from polyurethane foam that is designed to treat acute and chronic wounds with mild exudation. Biatain^®^ Alginate is an alginate dressing that is highly absorbent and designed for severely exuding wounds [18]. Mepilex Lite^®^ was chosen as the gold standard for all statistical studies because of its features and application [24].

##### Physicochemical Characterization

A physicochemical characterization of PF and polymers was carried out to evaluate the interactions, in a solid state, between pirfenidone and PLA-PCL copolymers. The solid-state characterization has been performed on raw materials and electrospun formulations.

##### Differential Scanning Calorimetry

The thermal features of PF, polymers, and the formulations were recorded using DSC (Mettler DSC821). Sealed samples were heated in aluminum pans, and an empty pan was used as a reference. In a nitrogen atmosphere with a 30 mL/min flow rate, heating curves were recorded in the temperature range of 25–180 °C with a heating rate of 3 °C/min.

##### X-ray Powder Diffraction

The crystallinity of the pure drug and the formulations was inspected using D2 PHASER—Bruker diffractometer (Milan, Italy). The XRD equipment was operated at 40 kV and 40 mA using Cu-Kα radiation in the range of (2θ) 5–60° with a scanning rate of 0.02°/s at room temperature, using the monochromatized diffractometer (λ = 0.154 Å) with graphite-sample monochromators. The crystallinity level was detected by applying the area integration method.

##### Morphological Characterization

The SEM pictures for the electrospun dressing (PL and Biofiber PF) characterization in terms of size, shape, and orientation of fibers were obtained using the Zeiss EVO MA10 apparatus (Carl Zeiss, Oberkochen, Germany). Dressing prototypes were scaled to be squares of 0.3 × 0.3 cm^2^; each sample was mounted on carbon stubs and covered with a gold layer, and then they were examined in high vacuum at room temperature at varied magnifications (50, 5.0, and 1.0 KX) and accelerated voltages (20 kV). ImageJ 1.44 software was used to evaluate all SEM pictures (*n* = 50). Digital vernier calipers were used to measure the diameter and thickness of the electrospun prototypes, as well as the weight [25].

##### Wettability Evaluation

The wettability behavior of the specimens (2 cm diameter) was assessed at 22 ± 3 °C and 36% relative humidity, in static drop modality. The contact angle meter (Kyowa Interface Science, Saitama, Japan, model: DMe-211Plus) was used for measurements, and the data were processed using FAMAS software (Version 3.3) from Kyowa Interface Science, Saitama, Japan. The drop’s contact time was set to 10 s. The hydrophilic solution used to evaluate dressing wettability was simulated wound fluid medium (SWF). To prepare SWF (100 mL), MRD powder was dissolved in 50 mL of distilled water to obtain a clear solution at a concentration of 9.5 g/L. Once the MRD solution had fully dissolved, it was mixed with 50 mL of FBS for 10 min. The results are expressed as average ± standard deviation (*n* = 3) [26].

##### Fluid Handling Capacity

The dressing’s ability to handle exudate and promote healing in a humid environment was represented by the fluid handling capacity (FHC), which was expressed as the sum of dressing absorbency and dehydration rate [5,18,27]. These characteristics were evaluated in accordance with the European standard BS EN 13726-1: Test Methods for Primary Wound Dressings [27]. The data are expressed as mean ± standard deviation (*n* = 3).

##### Absorbency of Dressing

The absorbency of the specimens was analyzed gravimetrically. The samples (2 cm diameter) were immersed in SWF and incubated for 24 h at 34 ± 2 °C, 30% RH. At fixed time points, samples were taken from the medium, and the fluid was drained for 60 s on absorbent paper. Equation (1) was used to calculate the absorbency of the dressing. The results are expressed as average ± standard deviation, *n* = 3.

(1) Absorbency %=(Wwet−Wdry)/Wdry

##### Moisture Vapor Transmission Rate

The moisture vapor transmission rate (MVTR) of the prototypes and relative controls (2 cm diameter) was measured in a laboratory oven for 24 h at 34 °C and 30% RH (Body PBI brand, VWR International Srl, Milano, Italy). After 24 h of conditioning, all samples were weighed (dry sample) and soaked for 30 min in an excess volume of SWF (2 mL). After 30 min, the samples were removed from the liquid and drained to remove any excess SWF. They were then weighed and dehydrated in a laboratory oven (34 °C, 30% RH) for 24 h to determine the rate of dehydration. Equation (2) was used to compute the results.

(2) Dehydration Rate (g/min)=W/(D/t)

where W is the sample mass (mg), D is the sample dry weight (mg), and t is the test time in minutes. The experiments were performed in triplicate (*n* = 3).

##### Vertical Wicking

The vertical wicking (VW) analysis of the dried samples (5.0 × 30 mm) was evaluated at 22 ± 3 °C and 36% RH. For 60 s, they were gently positioned vertically in SWF up to 10 mm in length. Using a straight edge, the VW measurement was calculated in millimeters. The results are expressed as the average ± standard deviation, (*n* = 3).

##### Mechanical Properties

To simulate the mechanical behavior of the dressings in contact with wound exudate, the tensile test was performed on Biofiber PF prototypes, Mepilex lite^®^, and Biatain^®^ Alginate specimens in moistureless conditions following incubation at 34 °C and 30% RH in SWF at fixed time points (24–72 h). A mechanical tester machine for uniaxial tensile tests (Mark-10 ESM303, Force Gauge Model MI5-5, G1013; Copiague, NY, USA) equipped with MESUR gauge Plus software (V2.1.0, Copiague, NY, USA) was used. The evaluations were carried out in accordance with the tensile properties of thin plastic sheeting (1 mm) guideline ASTM D882 (2002) [28]. Prototypes of standardized dog bone shapes (80 × 10 × 4 mm) were made using a calibrated die-cutting machine. The assessments were conducted following the ASTM D882 (2002) guideline for tensile properties of thin plastic sheeting (<1 mm). A calibrated die-cutting machine was used to obtain standardized dog-bone-shaped prototypes 80 × 10 × 4 mm in size. The analyses were performed at a constant 15.1 mm min^−1^ rate of tensile deformation. The measurements were used in determining the breaking point, yield strength, ultimate tensile stress (UTS), and Young’s modulus. The results are presented as the average difference between dry and wet conditions ± standard deviation, *n* = 10.

##### Dressing Integrity

To assess the electrospun prototype dressing integrity in wound exudate, a mass loss evaluation was carried out using an experimental protocol that mimicked the wound environment (34 °C and 30% RH in SWF, 24 h), [20]. Prototypes (about 10 mg, 2 cm in diameter) were incubated in exudate for 5 days before being removed and freeze-dried (Lio-5P, Cinquepascal, Italy) at predetermined intervals. The mass loss was assessed through gravimetric evaluation using Equation (3), [28].

(3) Mass Loss %=[(Wt−W0)/W0]×100

W0 is the initial weight of the sample, and Wt is the weight of the freeze-dried sample. The outcomes are presented as average ± standard deviation, *n* = 3.

#### 2.2.3. Drug Content and Encapsulation Efficiency

The drug content (DC) and the encapsulation efficiency (EE%) of pirfenidone (PF) loaded into the polymeric electrospun dressing fibers was obtained by dissolving Biofiber PF prototypes (10 mg) in 5 mL of DCM under magnetic stirring for 1 h in an ice bath. The EE% protocol was set up and validated by UV-Vis spectrophotometric (Jenway model 6705 scanning UV–visible spectrophotometer, Keison Products, Chelmsford, UK) at 310 nm. The PF concentration was determined using a standard calibration curve which was established starting from a stock solution containing 1 mg/mL PF in DCM, (Figure S1). The stock solution was diluted in a volumetric flask with DCM to obtain solutions of 5–30 μg/mL of PF. Each standard solution (1 mL) was analyzed in triplicate in high-precision cells made of quartz SUPRASIL^®^ (Hellma GmbH & Co. KG, Müllheim, Germany). Every data point on the calibration curve represents the mean of three separate analyses. The total DC of the pirfenidone-loaded dressings, expressed in μg/mg, and the encapsulation efficiency (EE%) of the drug into the electrospun fibers were calculated according to Equations (4) and (5), respectively:

(4) DC=MPF/Mdressing

where MPF is the mass of pirfenidone (μg) derived from the dressing, and Mdressing is the mass of the dressing (mg) from which the drug was recovered.

(5) EE%=(DC/Mtheor)×100

where DC is the amount of pirfenidone (μg) in the dressing and Mtheor is the amount of Pirfenidone (μg) added to the polymer solution (theoretically loaded) used to produce the advanced medicated dressing.

#### 2.2.4. In Vitro Release Study

To evaluate the electrospun dressing’s capacity for maintaining and regulating sustained release over a three-day period, an in vitro cumulative release of PF was assessed for 72 h. Samples (2 cm diameter) were weighted (16.83 ± 3.8 mg) and fixed into CellCrown™ (Sigma-Aldrich, Milan, Italy) inserts in 12 multi-wells (Sigma Aldrich, Milan, Italy). Next, 4 mL of phosphate-buffered saline 1× pH 7.4 (PBS 1×; Sigma Aldrich, Milan, Italy) was added to the respective well of each sample. The specimens were incubated in static conditions at 34 °C, 30% RH, in a laboratory oven. PF raw material (170 ± 32 µg) was used to compare Biofiber PF with a no-controlled release system. At scheduled times (2, 4, 6, 8, 24, 48, and 72 h), 700 µL of PBS was withdrawn from each well and assessed using an HPLC method taken from the literature [19] and adapted for pirfenidone quantification. Briefly, chromatography was carried out using a 1260 Infinity HPLC instrument (Agilent Technologies, Santa Clara, CA, USA) equipped with a Column Guard (C8, 4 × 30 mm) and a reverse phase LUNA column (C8(2), 5 μm, 100 Å, 250 × 4.6 mm) from Phenomenex Ltd. (Phenomenex Ltd., Aschaffenburg, Germany). An isocratic mobile phase consisting of acetonitrile 65% (ACN—isocratic grade for HPLC analyses, VWR International S.A.S, Rosny-sous-Bois-cedex, Milan, France) and 35% Milli Q water was used at a flow rate of 0.7 mL/min. The detection was carried out using a UV-Visible detector set at 310 nm. A calibration standard curve of PF in sterile PBS, ranging in concentration from 3 to 40 μg/mL, was calculated (Figure S2).

#### 2.2.5. Cell Culture

Normal human dermal fibroblasts (NHDF; Euroclone S.p.A, Pero, Italy) and hypertrophic scar-derived fibroblasts human (HSF, #HSF110 Lt Cheek; CellResearch Corporation, Singapore), were cultured in Dulbecco’s modified eagle medium, 1% *w*/*v* glutamine and 2% *w*/*v* sodium pyruvate (DMEM; Sigma Aldrich, Milan, Italy), and supplemented with 10% *v*/*v* FBS, 100 µg mL^−1^ penicillin, 100 µg mL^−1^ streptomycin (Immunological Science, Rome, Italy). They were maintained at 37 °C with 5% CO_2_. All experiments were performed using cells cultured within five to seven passages.

#### 2.2.6. Cytotoxicity Assay

An indirect thiazolyl blue tetrazolium bromide assay (MTT, approx. 98% TLC, Sigma Aldrich, Milan, Italy) was used to assess the biocompatibility of Biofiber PF dressing prototypes compared with NO-treated cells as a positive control (CTR+) and cells treated with phenol solution (Phenol Liquified 85% Re; Carlo Erba, Milan, Italy) as a negative cell viability control (CTR−), following the ISO 10993-12 guideline [29]: International Standardization Organization Biological Evaluation of Medical Devices. HSF cells were plated at a density of 10^4^ cells per well in Cellstar^®^ 96-well cell culture plates (Avantor VWR, Milan, Italy) and incubated under culture conditions for 24 h (37 °C with 5% CO_2_). Extracts of the electrospun dressings were prepared by incubating Biofiber PF round samples (2 cm diameter; 13.5 ± 2.6 mg) in DMEM (2 mL) for 24–48 h at 37 °C with 5% CO_2_. The pH of each extract was checked using an 827 pH lab pHmeter (Methron ion analysis Varese, Milan, Italy). No sign of massive degradation of PLA-PCL was detected, and the pH values ranged between 7.2 and 7.5. Cells were then incubated for 24 h with the specific extract, and then analyzed for the viability response [29]. Briefly, MTT powder was dissolved in PBS 1× (0.5 mg/mL), and the resulting solution was added to cells that were incubated for 3 h. Absorbance was measured at 570 nm using a microplate reader (HiPo MPP-96, OD plate (SIA Biosan, Riga, Latvia)) with 690 nm as the reference filter. The optical density value was directly proportional to the cell viability. Equation (6) was used to assess cell viability. Data are expressed as mean ± standard deviation (*n* = 9).

(6) Viability %=(Abs Sample/Abs Control)×100

#### 2.2.7. Live/Dead Staining

The morphological state of the cells and their viability was evaluated by means of Invitrogen LIVE/DEAD^®^ staining (Thermo Fisher Scientific, Milan, Italy). NHDF and HSF were seeded at a density of 5 × 10^4^ cells in Falcon 35 mm cell culture dishes (Avantor VWR, Milan, Italy) and grown for 24 h in 2 mL DMEM to avoid any stress condition after seeding. Cells treated for 72 h with Biofiber PF (65.45 ± 18.6 mg) extract or untreated cells (CTR) were stained with 500 μL of the L/D staining solution (1.5 mL of PBS 1×, 3 μL of EthD-1 and 1.5 μL of calcein). The samples were incubated at room temperature for 45 min in the dark, and after the removal of the LIVE/DEAD^®^ staining, cell nuclei were stained with 500 μL 4′,6-diamidino-2-phenylindole 1:1000 (DAPI; Sigma Aldrich, Milan, Italy) for 10 min. Fluorescence image acquisition was performed using a semi-confocal microscope (ViCo confocal, Nikon) [30]. The experiment was carried out with three replicates.

#### 2.2.8. DNA Synthesis by BrdU Incorporation

To understand the hypothetical regulation of cellular proliferation induced by the treatment of NHDF and HSF cells with Biofiber PF over time, a cell proliferation assay with 5-Bromo-2-deoxyuridine (BrdU, Sigma-Aldrich, Milan, Italy) was performed. Extracts of the electrospun dressings were prepared by incubating Biofiber PF (60.23 ± 20.3 mg) in DMEM supplemented with 10% FBS (2 mL) for 24–72 h at 37 °C with 5% CO_2_. The cells, seeded at a density of 5 × 10^4^, were cultured on DMEM growth medium supplemented with 10% FBS on previously autoclaved VistaVision™ microscope slides (Avantor, VWR International, Milan, Italy). When the cells reached semi-confluence, they were treated with 500 μL of Biofiber PF-specific extract for 24 h. The untreated cells were used as controls. The DNA synthesis was analyzed by measuring BrdU incorporation at the end of each treatment period. Briefly, the cells were labeled by adding 30 μM BrdU (Sigma-Aldrich, Milan, Italy) to the cell medium during the last hour of culture. Then, the cells were washed in PBS 1× and fixed in 70% ethanol (Carlo Erba, Milan, Italy). The incorporated BrdU was detected by immunostaining with anti-BrdU antibody (Sigma Aldrich, Milan, Italy). Briefly, the cells were washed with PBS and incubated in 2 N HCl (Carlo Erba, Milan, Italy) for 30 min at room temperature. Samples were neutralized in 0.1 M sodium tetraborate (pH = 8.5) for 15 min, washed twice in PBS for 5 min, and incubated for 20 min in PTA blocking solution (1% BSA and 0.02% Tween 20 in PBS 1×. The cells were then incubated for 1 h with mouse anti-BrdU antibody (diluted 1:100 in PTA) (Amersham Bioscience, Freiburg im Breisgau, Germany), washed three times in PTA, and then incubated for 30 min with anti-mouse FITC-conjugated antibody diluted 1:100 in PTA (Sigma-Aldrich, Milan, Italy). Then, the cells were extensively washed in PBS 1× and counterstained for DNA with 0.5 μg/mL Hoechst 33258 (Sigma-Aldrich, Milan, Italy). Finally, the cells were scored for immunofluorescence positivity with a fluorescence microscope (Zeiss Axiophot, Oberkochen, Germany), [31]. The experiment was carried out in three replicates. The percentage of proliferating cells was calculated using Equation (7):

(7) Cells proliferation=(Cells_s/Cells_tot)×100

where Cell_s indicates the number of cells in the S phase of proliferation, and Cell_tot refers to the total number of cells which were analyzed.

#### 2.2.9. Scratch Wound Assay

The scratch wound assay involved inducing empty space in a confluent monolayer of fibroblasts to simulate a wound. The protocol for a scratch wound assay typically consists of several key steps: cell culture preparation, scratch wound assay, data acquisition, and data analysis [32]. NHDF and HSF were seeded at a density of 5 × 10^4^ cells in a 12-well Cellstar^®^ cell culture plate (Avantor VWR, Milan, Italy) and grown in 2 mL DMEM until reaching semi-confluence. Biofiber prototypes (60.5 ± 4.1 mg) were sanitized under ultraviolet (UV) light for 24 h and fixed in the respective wells using sterile CellCrown™ inserts for 12-well plates to allow for treatment. Subsequently, the cells were treated with Biofiber PF for 24–72 h; non-treated cells were used as controls. At a specific experimental time point, a linear scratch “wound” was made in the center of a confluent monolayer using a 100 µL sterile tip. To monitor the cells filling the gap, images of the wounds were captured at regular time intervals (24–72 h) using a microscope Leica DM IL LED Fluo (Leica Microsystems S.r.l., Milan, Italy, equipped with a digital C–mount camera TP 5200 (Sony color CCD, Milan, Italy). The wound closure % and cell migration % were calculated by following Equations (8) and (9), respectively.

(8) Wound Closure %=[(t0−t1)/t0]×100

where t0 is the gap width before the treatment with Biofiber PF, and t1 represents the gap width after the treatment at specific experimental time points.

(9) Cell Migration %=[(t0−t1)/t]×100

where t0 is the gap width before the treatment with Biofiber PF, t1 indicates the gap width after the treatment at specific experimental time points, and t represents the time of treatment.

#### 2.2.10. RNA Isolation and Quantitative Real-Time PCR

HSFs were seeded at a density of 5 × 10^4^ cells/well in 12-well cell culture plates (Avantor VWR, Milan, Italy) and grown for 24 h in 2 mL DMEM. Biofiber PF matrices were cut into circular specimens (2 cm diameter ± 2.7 mg), sanitized using UV, and applied in the respective wells using sterile CellCrown™ inserts (Merck Life Science S.r.l., Milan, Italy) for 12-well plates to promote the treatment. Untreated cells were selected as the control (CTR). Biofiber PF treatment was conducted on the cells for 24–72 h in DMEM (2 mL); at scheduled experimental times, the inserts were withdrawn and the cells were collected for RNA isolation. Total RNA isolation was performed using 300 µL of Direct-zol RNA Miniprep’s reagents (Zymo Research; Euroclone S.p.A, Pero, Italy). The RNA concentration was assessed by NanoDropTM (Thermo-Fisher Scientific, Milan, Italy) at 260 nm. A total of 500 ng/µL of RNA was reverse transcribed using a iScript™ cDNA Synthesis Kit (Biorad, Milan, Italy), and quantitative real-time PCR analysis was conducted using the oligonucleotide primers reported in Table 1. The reaction was performed using MiniOpticon Real-Time PCR System (BioRad Laboratories, Milan, Italy), and data analysis was assessed using CFX Manager Software (V3.1). Gene expression was assessed in triplicate and normalized to glyceraldehyde 3-phosphate dehydrogenase (*GAPDH*) gene expression using the 2-DDCT formula. The antifibrotic effect of Biofiber PF was assessed by observing the gene expression profile of the major proteins involved in the fibrotic pathway: *TGF-β1*, *α-SMA*, tumor necrosis factor alpha (*TNF α*), *COL1A1*, and transforming growth factor β1 Receptor 1 (*TGF-β1R1*). Data are expressed as the mean ± standard deviation (*n* = 3).

#### 2.2.11. SDS–PAGE and Western Blot

HSFs were treated in the same conditions reported for the gene expression analysis, but to highlight evident changes in the structural protein components of treated cells, the treatment time was increased up to six days. HSF-treated and relative controls (CTR) were analyzed at 3–6 days for α-SMA protein levels by Western blot.

At a scheduled time, cells were collected and lysed with ice-cold lysis buffer (100 mM NaHCO_3_, 1 mM EDTA, 2% SDS, and 100 μM cocktail protease inhibitor, all from Sigma Aldrich, Milan, Italy) for 30 min on ice. The lysates were centrifuged at 13,000 rpm for 15 min at 4 °C, and supernatant protein concentrations were assessed using a bicinchoninic acid assay (BCA, Pierce Thermo Fisher Scientific, Parma, Italy) from a standard calibration curve, realized starting from a stock solution of 2 mg/mL of bovine albumin.

The samples (25 μg) were subjected to SDS-PAGE on 8% polyacrylamide gel; proteins were then placed onto Immun-Blot^®^ PVDF Membrane (BioRad Laboratories, Milan, Italy) and probed with primary anti-alpha-smooth muscle actin antibodies (1:100; Cell Signalling Technology, Euroclone SpA, Pero, Italy), followed by secondary antibodies conjugated to HRP (1:1000, Immunological Science, Rome, Italy).

Detection was achieved with enhanced chemiluminescence (ECL) reagents (Cell Signaling Technology, Euroclone SpA, Pero, Italy) and detected by autoradiography. The data are presented as mean ± standard deviation (*n* = 3), [33].

#### 2.2.12. Immunocytochemistry

To obtain cultures with visibly separable cells, NHDF and HSF were seeded in Falcon 35 mm cell culture dishes (Avantor VWR, Milan, Italy) at a density of 2000 cells/cm^2^ in complete media. Cells were grown for 24 h before the treatment with Biofiber PF for 6 days; untreated cells were used as controls. The samples were collected by fixing cultures in 4% paraformaldehyde in PBS 1× and storing in PBS 1× at 4 °C until all samples were ready to be stained. Fixed cells were then permeabilized via 10 min incubation in 0.1% Triton X-100 (Sigma Aldrich, Milan, Italy) in PBS 1×, then rinsed with PBS before blocking for 1 h in 5% normal donkey serum in PBT (PBS with 0.1% Tween 20; Sigma Aldrich, Milan, Italy). The cells were incubated for 1.5 h with the primary antibody anti-*α-SMA* (Sigma Aldrich, Milan, Italy), diluted at 1:50 in blocking buffer. Cells were rinsed with PBT before 1 h of incubation with Alexa Fluor 594-labeled goat anti-mouse secondary antibody (1:500; Immunological Science, Milan, Italy), Alexa Fluor™ 488 phalloidin (1:40; Immunological Science, Milan, Italy) and nuclear label 4′,6-diamidino-2-phenylindole 1:1000 (DAPI; Sigma Aldrich, Milan, Italy) for 10 min. Fluorescent image detection was performed using a semi-confocal microscope (ViCo confocal, Nikon, Amstelveen, The Netherlands), [34]. The experiment was carried out in three replicates.

### 2.3. Statistical Analysis

The data were presented as the mean ± standard deviation (*n* = 3). To analyze differences in the mean values between experimental groups, a two-way ANOVA (analysis of variance) was performed, followed by Tukey’s multiple comparison test, using GraphPad Prism 7.0 software. Significance levels were defined as * *p* < 0.05, ** *p* < 0.01, *** *p* < 0.001, and **** *p* < 0.0001.

## 3. Results

### 3.1. Advanced Dressing Characterization

The biofiber matrix was intentionally designed to offer advantages to both the patient and the clinician. Specifically, the prototypes are highly soft, flexible, and conformable to the skin when encountering fluids. They can easily conform to the shapes of awkward wounds and remain in contact with the wound surface. The textured surface promotes close contact of the dressing with the entire wound surface, thereby minimizing the potential for bacterial spread and surface colonization, (Figure S3).

The macroscopic characteristics of advanced dressings are described in Table 2. The variability in dimensions, thickness, and average weight is partly related to the composition of different formulations.

#### 3.1.1. Physicochemical Characterization

**Figure 1:**
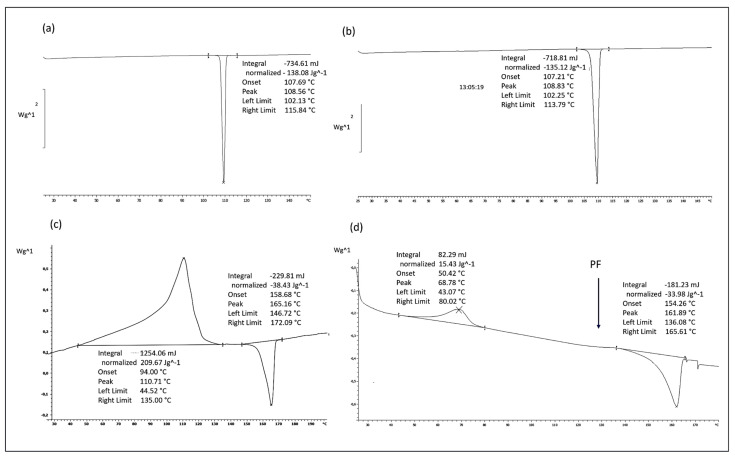
DSC heating curve of PF raw material (**a**), PF raw material after the cooling of the melt (**b**), PLA-PCL copolymer (**c**), and Biofiber PF formulation (**d**).

The DSC heating curve of PF shows a sharp melting endotherm at 108 °C, exhibiting its crystalline nature (Figure 1a). No evidence of an endotherm peak, nor of thermal curve variations, was highlighted as a sign that PF is stable, and it had no polymorphisms. During the cooling of the melt, an exothermic event of PF at about 34 °C (RT) and a subsequent melting peak at 108 °C during the heating run confirmed the product’s stability and the melting of the same crystal polymorphic form (Figure 1b). Until now, no other polymorphic forms have been detected. Regarding the PLA-PCL copolymer, the exotherm of recrystallization of amorphous material was recorded between 50 °C and 120 °C. This was attributed to the PCL polymer, which has a melting and softening point between 150 °C and 170 °C (Figure 1c). The DSC heating curve of PF-loaded electrospun formulations (Biofiber PF, Figure 1d) showed very similar thermal behavior of PLA-PCL, both in terms of temperature range and transition heat. No signal of PF was evident because of the low percentage of antifibrotic agents and a complete amorphous state in the Biofiber PF formulation (Figure 1d).

**Figure 2:**
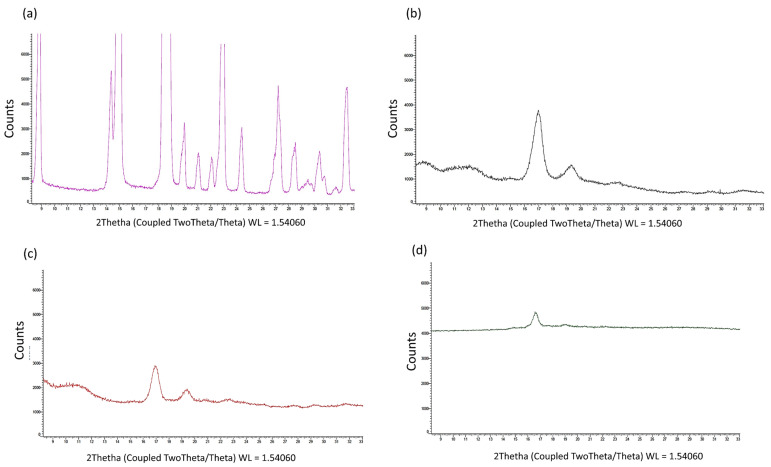
XRD powder patterns of PF raw material (**a**), PLA-PCL copolymer (**b**), Biofiber PLA-PCL from electrospinning process-placebo (**c**) and Biofiber PF formulation (**d**).

The X-ray analysis (Figure 2a–d) shows that (i) the copolymer PLA-PCL had typical diffraction peaks (17° and 19° 2-theta), indicating the presence of a weak crystalline phase from PCL [35]; (ii) the fused and recrystallized copolymer was completely amorphous, with an absence of diffraction lines; (iii) the PF was completely crystalline, with defined diffraction peaks characteristic of a compound with a high degree of crystallinity (Figure 2a).

PF-loaded electrospun formulations did not show the diffraction peaks of crystalline PF, but only a few characteristic peaks of the copolymer. The electrospinning process made PF completely amorphous and the copolymer much more amorphous. On the basis of this evidence, it was possible to assess that, during the electrospinning process, the solid matrix that was derived from the evaporation of the solvent system had no crystalline phase for the PF, but only traces for the PLA-PCL copolymer (Figure 2d).

#### 3.1.2. Morphology Characterization by Scanning Electron Microscopy

**Figure 3:**
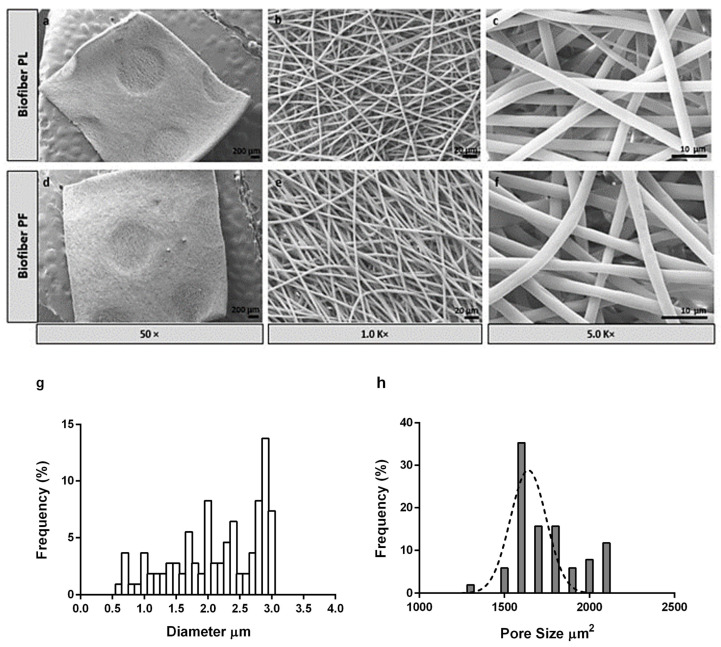
SEM images of placebo (Biofiber PL, (**a**–**c**)) and advanced medicated dressing loaded with pirfenidone 1.5% *w*/*w* (Biofiber PF, (**d**–**f**)) at different magnifications (50×, 1.0 K×, and 5 K×). (**g**,**h**) Graphical representations of Biofiber PF fibers’ diameter frequency %. (**g**) Histogram with a normal distribution of matrix pore size frequency % (**h**).

The SEM analysis of the sample revealed smooth and bead-less fibers with a well-interconnected matrix composed of uniform and random fibers. No significant differences were observed between the placebo (Biofiber PL) and the electrospun dressing loaded with Pirfenidone 1.5% *w*/*w* (Biofiber PF), proving that the addition of pirfenidone solution into the respective polymeric solution did not affect the morphology of the fibers. The average diameter of the fibers was 2.83 ± 0.46 µm, and the surface was smooth. Additionally, no evidence of PF crystals was observed by morphological analysis, either in the electrospun matrices or on the nanofiber surface (Figure 3).

The choice of the circular pattern was made after considering earlier research findings, which had shown its effectiveness in enhancing the mechanical characteristics of the dressing and its ability to conform well to the contours of the body’s surface. Additionally, this particular and bright texture contributes to wound transpiration and modulates the exudate [3,36]. To further enhance breathability, the electrospinning process was designed to control the pore size and distribution. The pores were distributed uniformly, with a size range of 1500–2000 μm^2^.

#### 3.1.3. Wettability Evaluation

**Figure 4:**
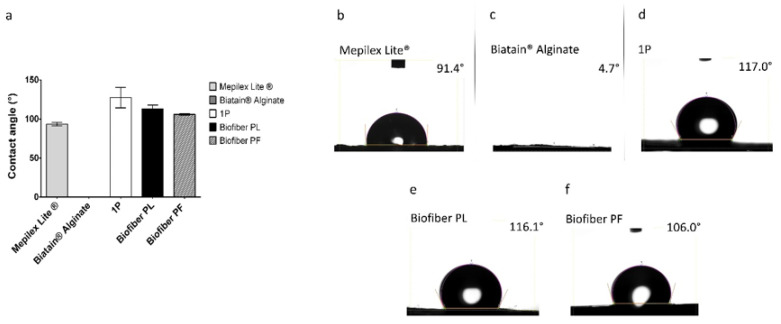
(**a**) Graphical representation of contact angles (°) related to Biofiber PF, Biofiber PL, and relative controls (Mepilex Lite^®^, Biatain^®^ Alginate and 1P); (**b**–**f**) representative images of contact angle assessment of each specimen considered in the analysis.

Samples of Mepilex^®^ Lite were observed to exhibit contact angle values of 93.43° ± 2.28°, which is consistent with the intended application of the medication. Biatain^®^ Alginate was found to manifest a null contact angle, as the SWF droplet was rapidly absorbed by the alginate component of the dressing. The non-textured electrospun dressing (1P) demonstrated solid hydrophobic behavior, with a wettability of 127.42° ± 7.13°. For the placebo samples (Biofiber PL), contact angle values of 112.77° ± 5.06° were reported, while the samples loaded with pirfenidone 1.5% *w*/*w* (Biofiber PF) exhibited values of 105.90° ± 0.99°. These values were slightly higher than those of Mepilex Lite^®^, but still within the same order of hydrophobicity, thereby confirming the appropriate wettability behavior of Biofiber PF. Moreover, compared to 1P, the wettability of the textured electrospun dressing was more suitable for the standard requisite of primary complex wound dressings [37,38]. Thanks to the circular pattern, Bofiber textured dressing is able to exhibit the correct wettability behavior and improve its exudate management. No statistically significant differences were observed between Biofiber PL and Biofiber PF. A two-way ANOVA test was conducted to assess the significance of the data between the samples and Mepilex Lite^®^ (Figure 4a).

#### 3.1.4. Fluid Handling Capacity

The fluid handling capacity (FHC) feature is the sum of the absorbency and permeability parameters and is indicative of the wound dressing’s ability to manage exudate. The evaporation of the aqueous component of the exudate increases the absorbency capacity and, consequently, the value of the FHC parameter. Effective exudate management helps to reduce the frequency of dressing changes, thereby avoiding disruption to the healing process and reducing treatment costs. The FHC feature was evaluated using the standard BS EN 13726-1 test [26].

The production of wound exudate is a fundamental aspect of the early inflammatory stage of wound healing. It is important to maintain an appropriate level of exudate for physiological healing, as an excessively dry wound bed can result in the formation of a scab, hindering wound contraction and desiccating the underlying collagen matrix and surrounding tissue at the wound’s edge [38,39]. Conversely, excessive exudate production can saturate the wound bed, causing moisture to leak onto the periwound skin and leading to maceration and excoriation [40].

**Figure 5:**
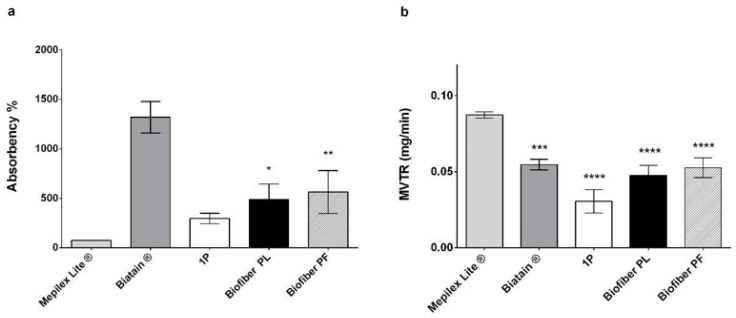
(**a**) Dressing absorption capacity (%) after 24 h of incubation in SWF (34 °C, RH Ambient). (**b**) Moisture vapor transmission rate (MVTR; 34 °C, 11% RH). Analyses were performed by comparing the placebo (Biofiber PL) and the medicated prototypes loaded with 1.5 *w*/*w* of pirfenidone (Biofiber PF) with those with no textured electrospun dressing (1P) and commercial controls (Mepilex Lite ^®^ and Biatain ^®^ Alginate). Statistically significant values are indicated as follows: * *p* < 0.05, *p* ** < 0.001 *** *p* < 0.001, **** *p* < 0.0001. A two-way ANOVA test was performed to evaluate the data significance between the samples and Mepilex Lite ^®^.

In this study, the absorbency of the electrospun medicated prototypes (562.90 ± 217.20%) was found to be intermediate between that of Mepilex^®^ Lite (73.72 ± 1.29%), a primary dressing for exuding wounds, and Biatain^®^ Alginate^®^ (1318.66 ± 159.52%), an alginate dressing for highly exuding wounds. This finding demonstrates the ability of electrospun dressings to maintain the proper balance of exudate within the wound bed (Figure 5a,b).

The MVTR is related to the chemical characteristics of formulations (composition) and the structural properties of dressings, such as dressing thickness and fiber orientation, diameter, and porosity [18]. The obtained results show that the highest MVTR values (between 0.08 and 0.055 mg/min) were obtained for Biatain^®^ Alginate and Mepilex^®^ Lite, respectively, which is consistent with the intended applications of these products. The electrospun placebo dressing (Biofiber PL) and that loaded with pirfenidone 1.5 *w*/*w* (Biofiber PF) demonstrated values like those of Mepilex^®^ Lite (ranging from 0.047 and 0.052 mg/min), indicating the promotion of an environment with an optimal moisture level for wound healing. No statistically significant differences were observed between the textured and non-textured electrospun dressings (1P; 0.031 ± 0.0076 mg/min).

Vertical wicking was utilized as a supplementary analysis method to deeply evaluate the effectiveness of the electrospun matrices in managing moisture. The analysis was conducted on advanced medicated dressing prototypes loaded with pirfenidone 1.5% *w*/*w* (Biofiber PF), as well as on the respective electrospun placebo (Biofiber PL). The results were compared with the controls (Mepilex Lite^®^, Biatain^®^ Alginate, and 1P). The vertical absorbent capacity of Mepilex Lite^®^ and Biatain^®^ Alginate was measured and was found to be 15 mm ± 0.33 and 31.6 mm ± 0.58 mm, respectively. These values confirmed that Mepilex Lite^®^ has moderate absorbent properties, while Biatain^®^ Alginate possesses the maximum absorbent capacity. The absorbent performances of both the NG-loaded and placebo electrospun samples were like that of Mepilex Lite^®^, resulting in 10.4 ± 3.81 mm and 10.1 ± 2.5 mm, respectively. No statistically significant result was detected between the textured electrospun prototypes and the untextured sample (1P, data not reported).

#### 3.1.5. Mechanical Properties

An advanced dressing that conforms to the skin’s surface and allows for patient movement can promote tissue oxygenation and angiogenesis, as well as preventing permanent contractures by avoiding prolonged immobilization of the affected area. These features are fundamental to improved wound healing and functional outcomes for the patient [38].

To assess the mechanical behavior of electrospun advanced dressings, a placebo (Biofiber PL) and a matrix loaded with pirfenidone 1.5% *w*/*w* (Biofiber PF) were analyzed in vitro by simulating a high-exudate environment through incubation in SWF at 34 °C and RH 50–60% for 72 h. By recreating a prolonged skin application environment, it was possible to examine the variation of Biofiber PF by comparing its mechanical properties in a “dry” state with those in a “wet” state at the end of application. The mechanical properties of the membranes were evaluated in terms of Young’s modulus, yield strength, ultimate tensile strength (UTS), breaking point, and elongation percentage at break.

**Figure 6:**
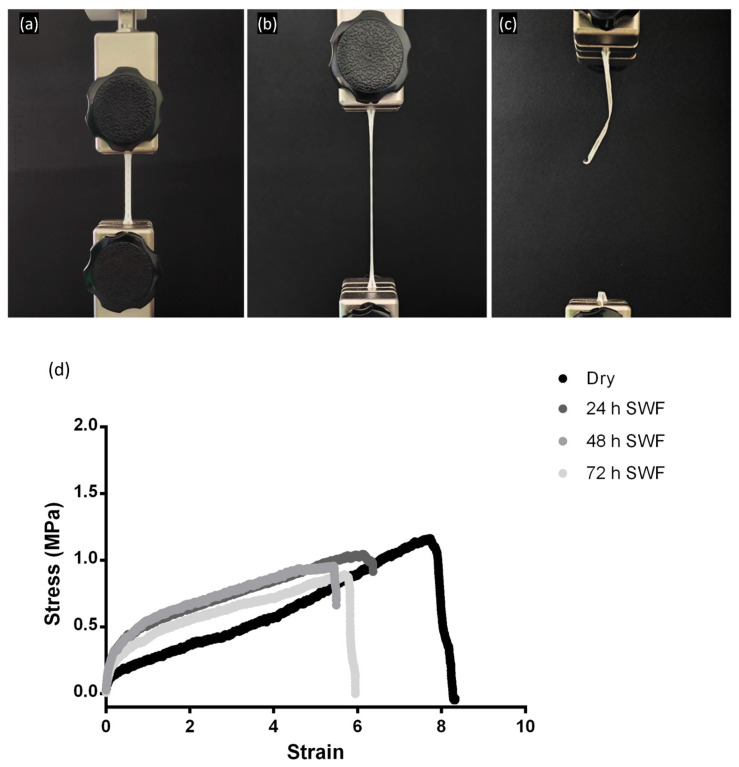
(**a**–**c**) Digital images of Biofiber PF prototypes during the testing of mechanical properties. (**d**) Representative tensile stress strain of Biofiber PF specimens in dry conditions and after 24–72 h in contact with SWF.

The mechanical properties of the biofiber were compared to those of Mepilex^®^ Lite and Biatain^®^ Alginate. During mechanical testing, an increase in applied stress resulted in the elongation of the samples due to thermoplastic elastomeric deformation. This type of plastic deformation is favorable, as it promotes matrix deformation while preserving its integrity under tension (Figure 6).

All formulations exhibited greater tensile strength than the commercial control Mepilex^®^ Lite, both in dry and exudate-soaked samples, for 24 h. Biatain^®^ Alginate showed limited mechanical properties with the minimum applied stress (data not reported). Both the Biofiber PL and Biofiber PF samples maintained their mechanical properties throughout the duration of the experiment, as no statistically significant changes were observed after 72 h of exposure to exudate (Table 3). The samples were analyzed in triplicate five times (*n* = 25).

#### 3.1.6. Dressing Integrity

The stability of a dressing is a fundamental requirement that must be evaluated to avoid and constrain the onset of any side effects of the treatment in the unstable physiological situation of a complex wound [16]. As reported in a previous study, a visual dispersion phenomenon was observed in the advanced electrospun dressing’s polymeric matrix over time in SWF [18], and the prototypes showed a minor mass loss of 8.0 ± 0.5%.

Regarding pirfenidone, as indicated in the regulatory assessment report for Esbriet^®^ (EMA/CHMP/115147/2011), pirfenidone is stable in a solid state under stress conditions. The photostability study demonstrated the good stability of PF in the solid state when exposed to visible light; however, degradation has been observed under visible light exposure of PF in solution [20].

### 3.2. Drug Content and Encapsulation Efficiency Evaluation

The overall calculated G EE% (global encapsulation efficiency percentage) of the electrospun medicated dressing (Biofiber PF) was 88.84 ± 10.30%, and the total drug content was 1.33 ± 0.154 mg per formulation (192.5 mg). These data highlighted the quality of the electrospinning process and the optimal result obtained in the dressing production process.

### 3.3. In Vitro Release Study

**Figure 7:**
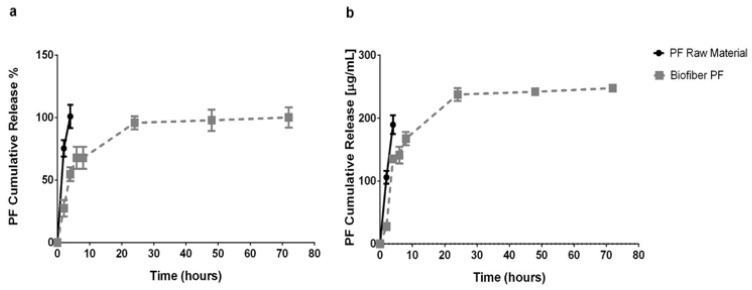
In vitro cumulative release, expressed as (**a**) pirfenidone cumulative release % and (**b**) pirfenidone cumulative release (µg/mL). The advanced electrospun dressing loaded with pirfenidone 1.5% *w*/*w* (Biofiber PF, 16.83 ± 3.8 mg) was incubated in PBS, pH 7.4, at 34 °C in static conditions. A dissolution of the respective powder was used as the control. The in vitro release was measured by HPLC analysis at 310 nm at 25 °C.

An in vitro release test was performed at scheduled experimental time points (2, 4, 6, 8, 24, 48, and 72 h) during the incubation of Biofiber prototypes (16.83 ± 3.8 mg) in PBS 1×, pH 7.4, at 34 °C in static conditions (Figure 7a,b).

This experiment was assessed in order to analyze the features of the innovative polymer’s fibrous medicated matrix (Biofiber PF) to achieve a 3-day controlled release of the encapsulated agent (Pirfenidone 1.5% *w*/*w*). The study aimed to address the challenge of obtaining prolonged antifibrotic action on the application sites of advanced dressings, thereby avoiding continuous dressing changes that can affect granulation tissue formation and cause discomfort to the patient [39].

The results showed that the pirfenidone loaded into the fibers achieved 50% of the sustained release in the first four hours and the rest over the course of three days; the burst release at the sixth hour reached 67.78 ± 8.85%. The high burst release was probably due to the small molecular dimension of pirfenidone (185.22 g/mol).

The results were compared with pirfenidone raw material to assess the difference between an uncontrolled system (raw material) and one with controlled release activity [21]. The dissolution of the PF powder (raw material) was completed in two hours, demonstrating the capability of Biofiber PF to promote a sustained and controlled release for three days.

### 3.4. Cytotoxicity Assay

The viability percentage of NHDF and HSF cells treated for 24–48 h with an extract derived from an advanced medicated dressing loaded with 1.5% *w*/*w* of pirfenidone (Biofiber PF) was assessed through an MTT assay. The viability of the treated cells was compared with the untreated cells as the positive control (CTR+), and with the cells treated with phenol as the negative control (CTR−). All samples were assessed in triplicate (*n* = 9).

**Figure 8:**
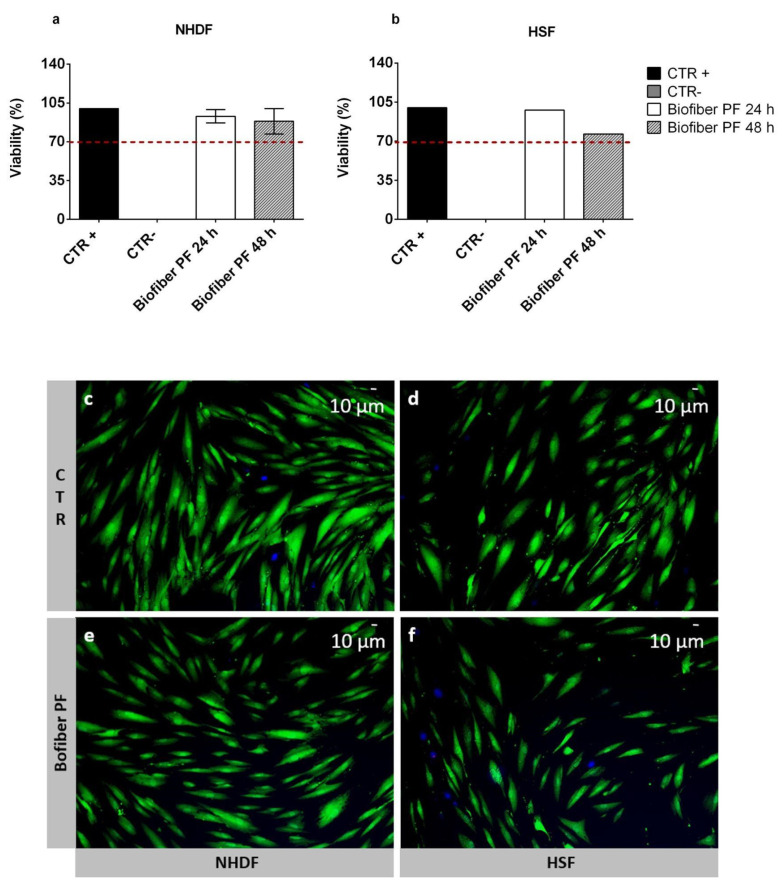
MTT assay of NHDF (**a**) and HSF (**b**) treated with Biofiber PF for 24 to 48 h; the results were compared with untreated cells (CTR+) and cells treated with phenol as negative controls (CTR−). The red dotted lines represent the viability threshold, fixed by following the ISO 10993-12 guideline. (**c**–**f**) Live/dead staining on NHDF and HSF untreated cells (**c**,**d**), as well as those treated with Biofiber PF extracts (65.45 ± 18.6 mg/2 mL DMEM) for 72 h (**e**,**f**). Green signal indicates live cells, and blue represents some of the marked nuclei. The detection was performed using a ViCo semi-confocal microscope (10×), the function of which was managed by means of IMAGE PRO 6.2 software (Houston, TX, USA).

The cell viability results were presented as the mean cell viability percentage ± SD. The highest viability was obtained at 24 h for both NHDF and HSF, at which point the values ranged from 93.94 ± 6.1% to 97.85 ± 9.2%, respectively. At 48 h, a decrease in viability was detected in both cell lines. However, the results were still above the viability threshold fixed at 70%, according to the ISO 10993 guideline [30]. For NHDF, the viability was 88.5 ± 11.5%, and for HSF, it was 76.48 ± 11.22%, (Figure 8a,b). This decrease in cell viability after 48 h of treatment was probably not due to a hypothetical cytotoxic effect of Biofiber PF, but rather to the ability of pirfenidone to attenuate fibroblast proliferation [21].

To further prove the biocompatibility of the electrospun matrix loaded with 1.5% *w*/*w* of pirfenidone (Biofiber PF), live/dead staining was performed on NHDF and HSF after 72 h of treatment. Untreated cells were used as controls (CTR).

In both cell lines, optimal viability was detected, and no evidence of cell damage or signs of apoptosis were observed (Figure 8c–f). Regarding the morphology of CTR cells, they were large, flat, and elongated (spindle-shaped), with a physiological shape (Figure 6c–d). As for the cells treated with Biofiber PF, the morphology was slightly different from the CTR, with lower dimensions and elongation in both cell lines which were considered, but no signs of alteration were detected. This result was probably due to the modulation of cell proliferation through the action of pirfenidone (Figure 8e,f) [22].

### 3.5. DNA Synthesis by BrdU Incorporation

**Figure 9:**
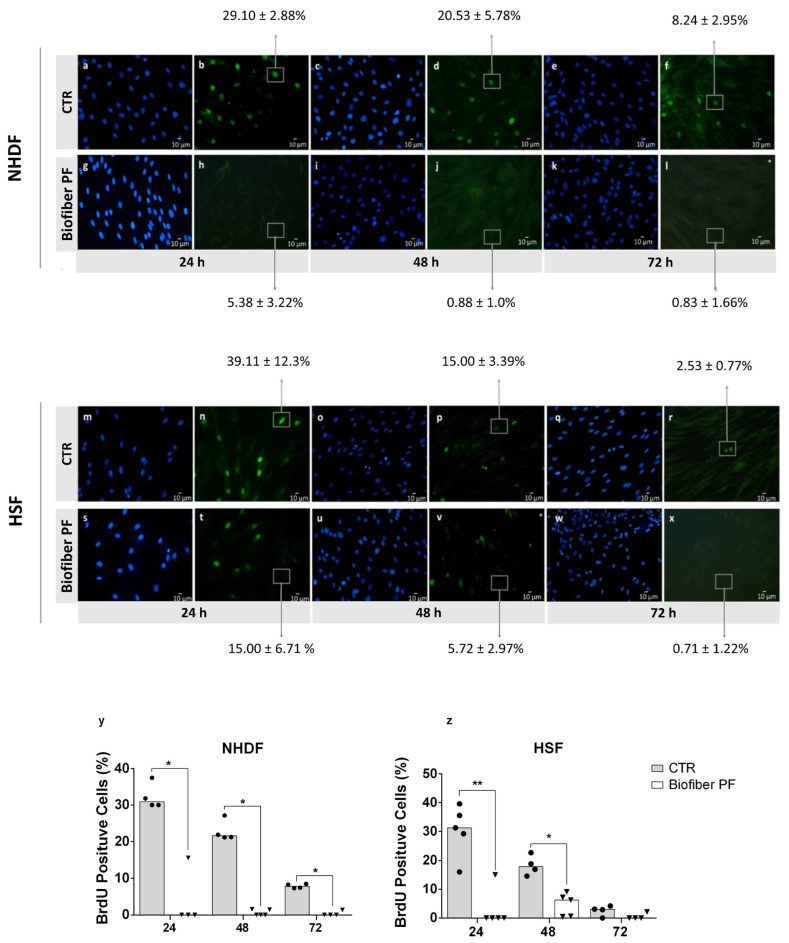
Proliferative activity of NHDF (**a**–**l**) and HSF (**m**–**x**), both untreated (CTR) and treated with Biofiber PF, for 24–72 h. Cell nuclei are visible in blue (Hoechst 33258), and proliferating cells stained for BrdU are visualized in fluorescent green (FITC-conjugated secondary antibody). Gray rectangles with relative arrows represent the percentages of cells with proliferative activity (number of cells displaying BrdU positivity compared with total counted cells). (**y**,**z**) Graphical representation of BrdU positive cells (%) untreated (CTR) and treated with Biofiber PF. Data are expressed as medians, and their respective distributions in the different examined replicates (*n* = 5) are represented by black circles (CTR) and triangles (Biofiber PF). Statistically significant values are indicated by * *p* < 0.05, ** *p* < 0.01. A one-way ANOVA (non-parametric Kruskal–Wallis test) was performed to evaluate the significance of the data between CTR and cells treated with Biofiber PF.

Bromodeoxyuridine (BrdU) incorporation assays have long been used to assess cell proliferation through DNA synthesis, both in vivo and in vitro. The key principle of this method is that BrdU, incorporated as a thymidine analogue into nuclear DNA, represents a label that can be tracked using antibody probes [40,41]. In this study, BrdU incorporation into NHDF and HSF cells was used to assess the effect of pirfenidone loaded into the electrospun fibers of Biofiber PF (pirfenidone 1.5% *w*/*w*) on the modulation of cell proliferation (Figure 9). Untreated cells (CTR) and cells treated with Biofiber PF were analyzed at specific experimental time points (24–72 h).

In the case of NHDF, a significant downregulation of cell proliferation was clearly visible at all time points analyzed in cells treated with Biofiber PF (Figure 9a,c). However, in HSF, the modulation of DNA synthesis was significant at 24 and 48 h of treatment, with no significant difference observed at 72 h due to the state of confluence of cells in both experimental conditions (Figure 9b,c).

The difference in the response of NHDF and HSF (Figure 9m,x) to the treatment was likely due to the higher rate of replication of HSF cells compared to NHDF cells. This difference in replication rate is one of the major causes of the thickening of hypertrophic scars and keloids [42].

For each experiment, 3 × 10^2^ cells were analyzed to detect the proliferation process, and the samples were analyzed in triplicate (*n* = 5).

### 3.6. Scratch Wound Assay

When a wound occurs, skin cells start to proliferate and migrate over to the wound bed in order to close the wound. Migration is an important process that influences several physiological aspects. In wound healing, migration facilitates the re-epithelization of the skin. Unfortunately, in complex wounds subject to fibrosis, the rate of cell migration appears to be upregulated, and when combined with hyperproliferation, it can lead to an irregular wound closure process [43].

**Figure 10:**
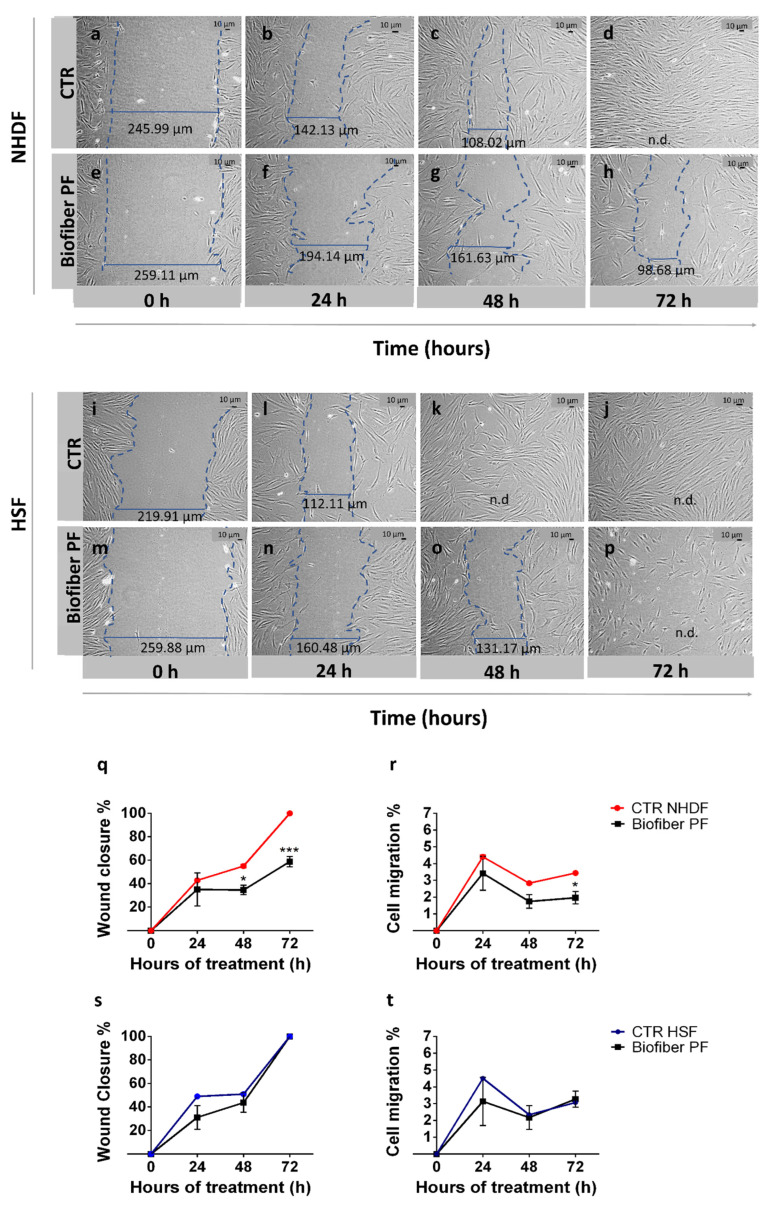
Wound healing scratch assay performed on NHDF (**a**–**h**) and HSF (**i**–**p**) cells, both untreated (CTR) and treated with the advanced medicated electrospun dressing loaded with pirfenidone 1.5% *w*/*w* (Biofiber PF), for 24–72 h. The blue dotted lines indicate the wound edges, and the horizontal lines represent the measurement of the wound gap over time. Percentages of wound closure (**q**,**s**) and the rate of cell migration (**r**,**t**) in a 2D scratch assay are shown, comparing the results obtained from untreated cells (CTR) and cells treated with the advanced medicated dressing containing 1.5% *w*/*w* Pirfenidone (Biofiber PF) at different experimental times. The red and light blue lines refer to NHDF and HSF untreated, respectively; statistically significant values are indicated as * *p* < 0.05, *** *p* < 0.001. A two-way ANOVA test was performed to evaluate the significance of the data between CTR and cells treated with Biofiber PF.

In this study, the scratch wound assay was used to analyze the differences in wound closure % and cell migration % of NHDF and HSF in a 2D experimental injury, which was either treated with Biofiber PF for 24–72 h or not (CTR) (Figure 10a,b).

### 3.7. Gene Expression Analysis by Quantitative Real-Time PCR

Gene expression analysis was performed using qPCR at 24–72 h after treatment with Biofiber PF to validate the antifibrotic effect of pirfenidone 1.5% *w*/*w* as a therapeutic antifibrotic agent for complex wounds. Human skin fibroblasts (HSFs) were chosen as suitable candidates to represent a wound healing process similar to a fibrotic phenotype. All of the results were compared with the respective controls. The data reflect the results from three separate and distinct experiments.

The gene expression of the major fibrotic proteins, such as *TGF-β1*, *α-SMA*, and *COL1A1,* was detected by RT-qPCR normalized by the *GAPDH* gene expression. After 24 h of Biofiber PF treatment, no significant downregulation of any of the considered genes was observed. This was likely due to the function of *TGF-β1*, *α-SMA*, and *COL1A1*, which are constitutive proteins strongly expressed in fibroblasts or in the connective matrix, and a treatment lasting only 24 h might not be optimal for assessing a significant effect. However, after 48 h of treatment with Biofiber PF, a preliminary antifibrotic action could be assessed. The expressions of *TGF-β1* and *α-SMA* genes were 2.89 and 3.23-fold lower, respectively, in HSF treated with Biofiber PF with respect to the untreated cells (*p* < 0.05, *p* < 0.001). In addition, the *COL1A1* gene was downregulated by 1.98-fold compared to the untreated cells. However, considering the other experimental time points, this effect could be attributed to a slightly consistent modulation exerted by the effect of the pirfenidone released by the polymeric fibers of Biofiber PF. *COL1A1* is a protein that plays a crucial role in the structural composition of the connective tissue in wounds, and its expression is associated with the formation of scars. However, modulating its expression requires treatment for an extended period of time. In this analysis, the effects of Biofiber PF treatment on the modulation of *COL1A1* expression were only partially observed due to the limitations of the in vitro cell culture model in the experimental timeline.

On the third day of treatment, the gene expression of *TGF-β1* and *α-SMA* was slightly modulated; the levels of expression of *TGF-β1*, and *α-SMA* were, respectively, 0.83- and 0.71-fold lower than the untreated cells, and no statistically significant results were detected for *COL1A1* gene expression, even though the downregulation of the three genes persisted (*p* < 0.05, *p* < 0.001), Figure S4. The data are presented as mean ± standard deviation (*n* = 3).

### 3.8. Western Blot and Immunocytochemistry

Severe scar contracture, which is associated with hypertrophic scars, often results from complex injuries and is a major issue for burn survivors, particularly pediatric burn victims [44]. Scar contracture is generally believed to be caused by the presence of elevated numbers of myofibroblasts in scar tissue, which express α-smooth muscle actin *(α-SMA*, 42 kDa). *α-SMA* affects scar contraction formation and is usually found only in the vessel wall and arrector pili muscle in normal skin tissue [45]. Following a burn injury, myofibroblasts become the dominant cell type in granulation tissue [46].

Because of the important role of *α-SMA* in hypertrophic scar formation, the antifibrotic activity of an advanced medicated dressing was assessed by focusing on a preliminary analysis of the levels of this element in untreated hypertrophic scars (CTR), as well as those treated with Biofiber PF, for 3–6 days.

**Figure 11:**
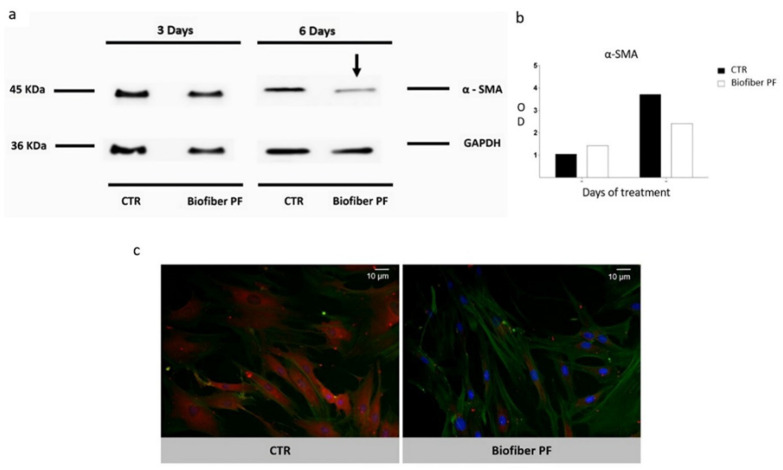
(**a**) Representative Western blot results of α-SMA (42 kDa) modulation in HSF, both untreated (CTR) and treated with Biofiber PF for 3 and 6 days. Black arrows highlight relevant results. (**b**) Graphical representation of *α-SMA* blot bands of plots normalized against total *GAPDH* expression. (**c**) Immunocytochemistry of HSF, both untreated (CTR) and treated for 6 days with Biofiber PF. Cells were stained for *α-SMA* (red), nuclei Hoechst (blue), and F-actin (green). The observation was performed using a semi-confocal microscope (20×), the function of which was managed by IMAGE PRO 6.2 software. Scale bar represents 100 μm.

The results, reported in Figure 11a, show a time-dependent modulation of protein levels, with *α-SMA* blot bands normalized against total *GAPDH* expression. After 3 days of treatment, no significant results were detected. The most significant result was observed after 6 days of treatment, with a modest decrease in *α-SMA* protein levels. Considering the gene expression results, the delay in *α-SMA* regulation was probably due to the consistent presence of this protein as a part of the cytoskeleton and to the delay in the process between gene transcription and protein translation.

Western blot analysis was confirmed through immunocytochemistry staining specifically for *α-SMA* detection (red signal). To understand any hypothetical variations in cell connection, F-actin was assessed by phalloidin staining (green signal). According to the Western blot results, a decrement in *α-SMA* protein expression was detected in HSF treated with Biofiber PF for 6 days.

Analyzing the cell morphology, the cells treated with Biofiber PF for 6 days showed a smaller and less compact structure compared to the controls. This characteristic was also observed, albeit to a lesser extent, during the live/dead and scratch assay at 72 h. However, no significant variations were observed in the cytoskeletal connections between cells, which remained solid and well-connected even after treatment (Figure 11c). The data are presented as mean ± standard deviation (*n* = 3).

## 4. Discussion

An advanced medicated dressing loaded with pirfenidone 1.5% *w*/*w* was produced using an electrospinning process based on patented technology [18]. The electrospinning process is an innovative technique that enables the production of fibrous structures with a considerable surface area, not only for cell attachment, but also for oxygen permeation and fluid management [17,18]. The success of wound dressings fabricated by electrospinning is attributed to their microporous structure, with fibers having diameters ranging from the nano- to the micro-scale [47,48].

Furthermore, the features of this new generation of advanced wound dressings can be optimized through the selection of the most suitable biomaterials for fabrication [17,49]. Numerous studies in the scientific literature have demonstrated the complementarity of PLA with PCL. The combination of these two polymers as copolymers or polymer blends enables the production of materials with the desired characteristics of both constituent elements. PLA is renowned for its stability and robustness, while PCL is widely used in wound healing products due to its intrinsic tissue adhesion and excellent conformability [3,17,18,50].

The enormous potential of these innovative materials is evident from the numerous patents and successful products that have already captured the attention of the wound care industry. Their unique properties and versatility make them highly sought after and pave the way for exciting new possibilities in the field of wound healing [9,42]. Such materials may offer new opportunities for the development of advanced wound care products with improved performance and functionality. These developments may have significant implications for the healthcare industry, including the potential for improved patient outcomes and reduced healthcare costs [51].

In this study, a comparative analysis was conducted to evaluate the characteristics of Biofiber PF, an electrospun textured dressing loaded with 1.5% *w*/*w* of pirfenidone, compared to Biofiber PL (placebo electrospun textured dressing), 1P (electrospun untextured dressing), Mepilex Lite^®^, and Biatain^®^ Alginate (commercial polyurethane-based and alginate-based dressings, respectively). Criteria such as fluid handling management and mechanical properties were used for the assessment [27,52].

This study found that there were no significant differences between Biofiber PF and its placebo (Biofiber PL) in either of the parameters that were assessed, which indicates that the addition of the antifibrotic agent did not have an impact on the behavior of the electrospun formulation. However, important differences were observed when comparing Biofiber PF to the untextured dressing and commercial products, especially in terms of moisture balance. The circular textures of the Biofiber prototypes were found to improve all aspects related to exudate management compared to untextured electrospun prototypes. This is consistent with previous studies that have shown that the textured prototypes have unique properties that are crucial to preventing the risk of maceration and eschar formation [3,18]. Biofiber PF exhibited similar fluid-handling characteristics to Mepilex Lite^®^, one of the most widely used dressings for complex wounds, but with some advantages regarding exudate absorption. Mepilex Lite^®^ is known for its resistance and conformability properties, given its polyurethane-based composition, but unfortunately has poor absorption properties. In contrast, Biofiber PF demonstrated an absorption capacity that fell between that of Mepilex Lite^®^ and the excessive absorption observed in Biatain^®^ Alginate. This aspect represents the key success factor of Biofiber compared to other dressings. This new generation of dressings can create healing environments with calibrated and specific moisture levels to prevent pathological wound progression.

In addition to exudate management, one of the main demands from clinicians and wound care experts is the development of dressings with important mechanical properties and specific properties for body areas that are often difficult to manage, such as the lips, neck, and curved areas [53,54]. Conformability and resistance are crucial properties in the functional recovery of patients because they allow for movement, and, consequently, tissue oxygenation and prevention of contractures [55,56]. Polyurethanes, such as Mepilex Lite^®^, have excellent elastomeric and resistance properties, but tend to preserve their shape, demonstrating poor adaptation to body surfaces. Alginate-based dressings, such as Biatain^®^ Alginate, have poor mechanical properties and, due to their fragility, were not evaluable in this study. For the Biofiber prototypes [3,11], the electrospinning time was set at 20 min to further implement the mechanical behavior of its polymer matrix over a period of time in SWF. Biofiber PF demonstrated optimal elongation and tenacity properties due to its thermoplastic elastomeric profile. This behavior is typical of PLA-PCL-based products and represents a fundamental feature with which to obtain excellent conformability during the application time; moreover, the adaptation to the body surface is further enhanced by the circular pattern of the Biofiber PF texture [3,18,48]. According to the stability data, no statistically significant variation was observed in the behavior of this electrospun matrix under dry conditions or after 72 h of contact with the exudate [18]. This structural maintenance is essential for a dressing intended to remain on the wound bed for 3 days.

Numerous studies have investigated the properties and behavior of PCL-based polymers in drug delivery, exploring their ability to encapsulate and release drugs in a controlled manner [57,58,59]. These studies have shown that PCL and its copolymers can serve as effective drug carriers, improving the efficacy and safety of drugs by delivering them directly to the target sites and controlling their release rate. The results of in vitro cumulative release studies have shown that PLA-PCL electrospun textures fibers can allow the sustained controlled release of pirfenidone for a period of three days. These features, along with its stability and exudate management, confirmed its sustained functional antifibrotic performance on the wound bed. By enabling prolonged drug release, Biofiber PF contributes to the reduction in dressing changes and the resulting decrease in healthcare expenses. Additionally, this feature enhances patient compliance by minimizing the need for frequent wound care procedures.

According to the controlled release results, the antifibrotic activity of Biofiber PF was analyzed in order to verify the regulation of cell proliferation and major scarring genes through the released pirfenidone for 72 h.

Complex wounds are heterogeneous environments characterized by the presence of non-pathological dermal cellular elements (NHDF) and cells tending toward scar formation (HSF) [60]. Moreover, in a complex wound, hyperproliferation of cells and an imbalance of collagen deposition are one of the major causes of hypertrophic scars; therefore, it is important to facilitate wound closure while maintaining adequate cell proliferation and migration and constraining the major factors causing the fibrotic phenotype [6,61].

After simulating a complex wound in vitro by means of a scratch assay, the ability of Biofiber PF to modulate the proliferation and migration was observed on NHDF dermal cells at different time points (24–72 h). One of the important features of this dressing is its ability to promote wound closure while regulating cell proliferation, thereby achieving physiological healing and preventing the formation of fibrotic contractures caused by abnormal cell proliferation. Unfortunately, the same effect was not achieved on established scar tissue cells (HSF). The structural morphology and intrinsic behavior of the cells were not found to be modulated in terms of proliferation using Biofiber PF as the treatment.

To investigate the consistent action of Biofiber PF in terms of inhibiting the fibrotic phenotype in complex wounds, this study focused on analyzing the gene expression of fibrotic proteins, including *TGF-β1*, *alpha-SMA*, and *COL1A1.* Furthermore, the study evaluated the modulation of alpha-SMA protein levels, which are a major contributor to the fibrotic process in complex wounds and a target of pirfenidone [3,20]. The HSF cell line was selected as the target for assessing the therapeutic action of Biofiber PF in a healed wound [62].

Biofiber regulates the gene expression genes of *TGF-β1* and *alpha-SMA* through the PF-controlled release for 72 h. Contrary to other studies, in this research work, only a slight modulation of *COL1A1* gene expression was evidenced [63]. *COL1A1* is a high-molecular-weight structural protein (120 KDa) that is densely expressed in the connective matrix of fibrotic tissue. The modulation of its expression is important in wound healing, but due to its fibrous structure and abundance in connective tissue, it requires extended treatment over time. Therefore, due to the limitations of in vitro cell culture experimental timelines, the modulation of *COL1A1* in vitro after treatment with Biofiber PF could only be partially observed. Observing the effects of Biofiber PF on *COL1A1* modulation through in vivo experimental models, which allow for the continuous application of Biofiber PF over time, is necessary in order to confirm the effectiveness of this advanced medication in modulating COL1A1. Positive findings related to the decrease in α-SMA protein levels were exclusively seen after 6 days of administering Biofiber PF, with no notable outcome observed after 3 days. This discrepancy in the timing of gene expression and protein level results was attributed to the time lapse between gene transcription and protein translation. Due to the constraints of the in vitro experimental model, the analysis of protein levels could only be conducted in a representative manner. However, considering the vital structural role played by both proteins, it is plausible to assume that a considerable reduction could be achieved with continuous application of Biofiber throughout the wound healing process.

Pirfenidone is the first antifibrotic drug to be approved for the treatment of idiopathic pulmonary fibrosis (IPF), and it is used in the treatment of other interstitial pneumonias, such as unclassifiable interstitial lung disease (ILD) and connective-tissue-related ILD [62,64]. The action of these antifibrotic agents in the wound healing field is a source of interest within the scientific community [65,66,67]. In 2018, it was demonstrated that pirfenidone ointment performs its wound healing action by providing a positive response, modulating the inflammation in a burn skin murine model [68]. However, pirfenidone as an antifibrotic agent in complex wounds still needs to be investigated [69].

## 5. Conclusions

This study utilized the diverse physical, chemical, and mechanical properties offered by electrospinning technologies to showcase the potential of Biofiber PF as a significant scientific contribution to the wound care field. The innovative fibrous matrix of Biofiber PF offers numerous advantages, including enhanced conformability and resilience to the application site, as well as an extended release of the antifibrotic agent for up to three days. This sustained effect on the wound bed is crucial in minimizing damage to the granulation tissue, alleviating pain, and ultimately improving healing outcomes and patient compliance.

The real strength of Biofiber PF, however, lies in its exceptional potential as a therapeutic antifibrotic treatment. When applied throughout all phases of the wound healing process, Biofiber PF could consistently exhibit antifibrotic effectiveness, making it a more advantageous option when compared to traditional invasive treatments.

To further validate the effectiveness of Biofiber PF, ongoing ex vivo studies are currently being conducted to evaluate its efficacy in the connective reorganization of healing tissue, particularly in complex environments. These studies, combined with forthcoming in vivo studies on appropriate experimental scarring models, will be pivotal in fully validating the extraordinary potential of Biofiber PF as a topical prophylactic antifibrotic treatment.

## Figures and Tables

**Table 1:** Summary of quantitative PCR analysis primers.

Gene	Primer Forward	Primer Reverse
*H. sapiens TGF-β1*	5′-GGACCAGTGGGGAACACTAC-3′	5′-GGCATGGACTGTGGTCATGA-3′
*H. sapiens α-SMA*	5′-GCAGCCGAGCCAAGCACTGT-3′	5′-TGGGAGCATCGTCCCCAGCA-3′
*H. sapiens COL1A1*	5′-CTGCCTGGTGAGAGAGGTC-3′	5′-CACGATGACCACGACGGC-3′
*H. sapiens GAPDH*	5′-TTCACCACCATGGAGAAGGC-3′	5′-GGCATGGACTGTGGTCATGA-3′

**Table 2:** Placebo (Biofiber PL) and electrospun prototypes loaded with Pirfenidone 1.5% *w*/*w* (Biofiber PF); macroscopic characterization of weight, height, length, and thickness. Measurements are expressed as the mean value ± standard deviation.

Sample	Weight (mg)	Height (mm)	Length (mm)
Biofiber PL	168.2 ± 18.8	54.4 ± 5.7	106.4 ± 5.6
Biofiber PF	203.2 ± 3.0	39.1 ± 5.5	120.9 ± 8.7
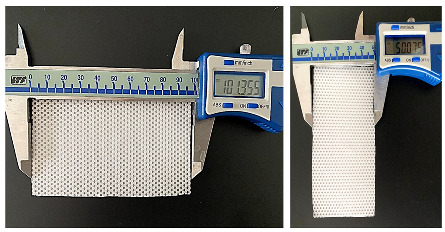			

**Table 3:** Biofiber ΔL% mechanical properties of dry vs. wet sample conditions (SWF, 34 °C for 72 h).

Samples	ΔL%				
	Elongation (%)	UTS (Mpa)	Breaking Point (Mpa)	Yield Strength (Mpa)	Young’s Modulus (Mpa)
Biofiber PL	−0.243 ± 0.01	−0.80 ± 0.69	−0.807 ± 0.67	−0.235 ± 0.23	−1.02 ± 1.3
Biofiber PF	−0.207 ± 0.02	−0.42 ± 0.18	−0.180 ± 0.03	−0.455 ± 0.39	−0.11 ± 0.34

## Data Availability

Data will be made available on request.

## Supplementary Materials

The following supporting information can be downloaded at: https://www.mdpi.com/article/10.3390/polym15204045/s1, Figure S1: UV-Vis spectrum of Pirfenidone standard solution in PBS 1× pH 7.4 (20.0 μg/mL) (a). Pirfenidone UV-Vis calibration curve at different concentration (5–30 µg/mL) measured at 310 nm (b). Figure S2: Calibration curve of PF solutions of different concentrations (3.0–40 µg/mL) measured at 310 nm at 25 °C. Figure S3: Optical microscope images at different magnifications (4, 20 and 20×) of placebo (Biofiber PL, a–c), and advanced medicated dressing loaded with Pirfenidone 1.5% *w*/*w* (Biofiber PF, d-f). Figure S4: Gene expression analysis of HSF treated with Biofiber for 24, 48 and 72 h. (a) qRT-PCR at 24 h. (b) qRT-PCR at 48 h. (c) qRT-PCR at 72 h.
