# Supplementary material for: Electrospun Fibers Loaded with Pirfenidone: An Innovative Approach for Scar Modulation in Complex Wounds
Source: Polymers (Basel). 2023 Oct 10;15(20):4045. doi: 10.3390/polym15204045 (PMC10610295; doi:10.3390/polym15204045)
Supplement: Supplementary file 1 [file polymers-15-04045-s001.zip › polymers-2616760-supplementary.pdf]

polymers

MDPI

Supplementary Materials

Electrospun Fibers Loaded with Pirfenidone: An Innovative Approach for Scar Modulation in Complex Wounds

Erika Maria Tottoli [1], Laura Benedetti [2,3], Federica Riva [4], Enrica Chiesa [1], Silvia Pisani [1], Giovanna Bruni [5], Ida Genta [1,3], Bice Conti [1,3], Gabriele Ceccarelli [2,3] and Rossella Dorati [1,*]

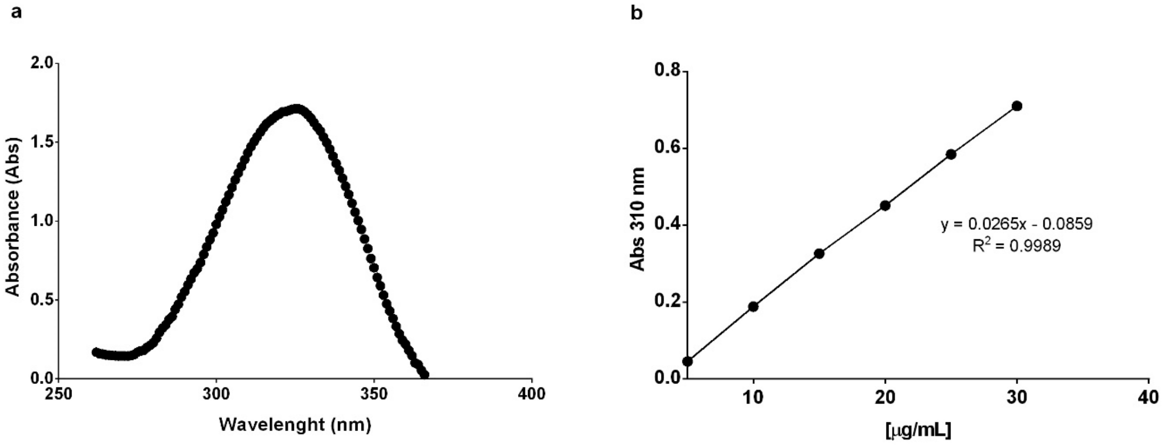

Figure S1. UV-Vis spectrum of Pirfenidone standard solution in PBS 1 X pH 7.4 (20.0 µg/mL) (a). Pirfenidone UV-Vis calibration curve at different concentration (5-30 µg/mL) measured at 310 nm (b). Standard deviations are not noticeable as <0.01, plot b.

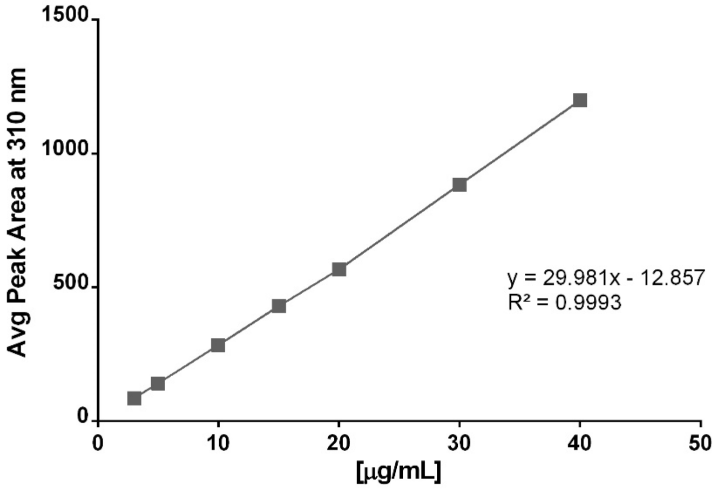

Figure S2. Calibration curve of PF solutions of different concentrations (3.0–40 µg/mL) measured at 310 nm at 25°C. Standard deviations are not noticeable as < 5.00.

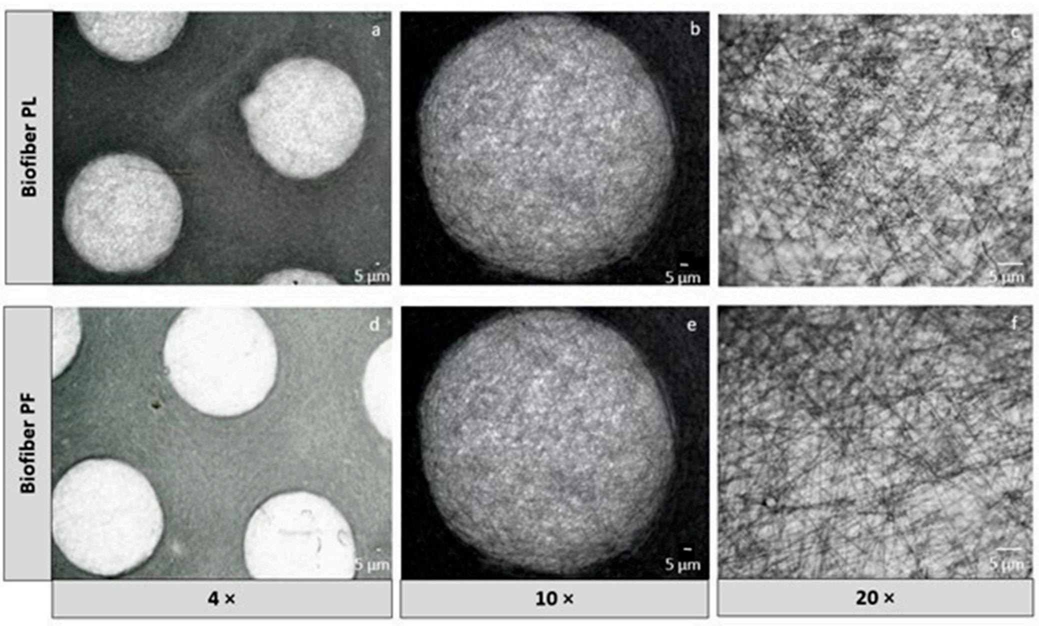

Figure S3. Optical microscope images at different magnifications (4, 20 and 20 ×) of placebo (Biofiber PL, a-c), and advanced medicated dressing loaded with Pirfenidone 1.5% w/w (Biofiber PF, d-f).

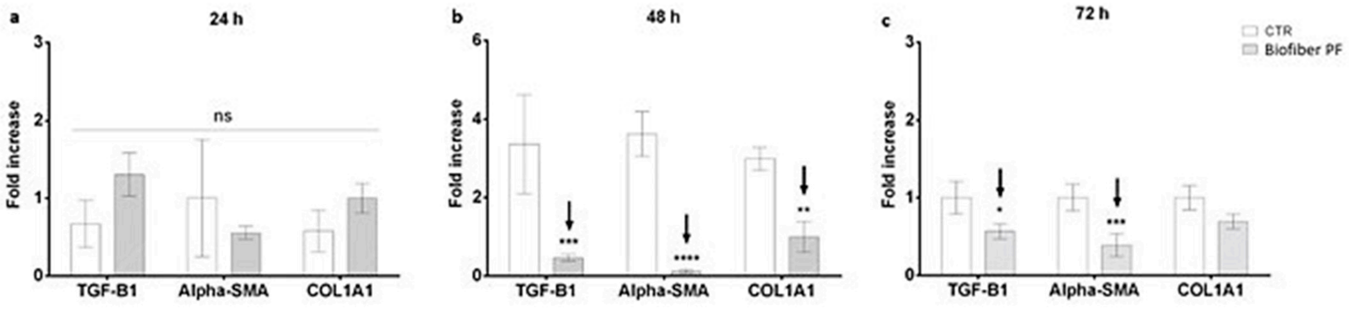

Figure S4. Gene expression analysis of HSF treated with Biofiber for 24, 48 and 72 h. (a) qRT-PCR at 24 h. (b) qRT-PCR at 48 h. (c) qRT-PCR at 72 h. Results are normalized to the housekeeping gene (Glyceraldehyde-3-Phosphate Dehydrogenase (GAPDH)). Statistically significant values are indicated as * $p < 0.05$, ** $p < 0.01$, *** $p < 0.001$, **** $p < 0.0001$. Analysis of variance test was performed to evaluate data significance.
